# *Lactobacillus reuteri* 5454 and *Bifidobacterium animalis* ssp*. lactis* 5764 improve colitis while differentially impacting dendritic cells maturation and antimicrobial responses

**DOI:** 10.1038/s41598-020-62161-1

**Published:** 2020-03-24

**Authors:** Jiří Hrdý, Jeanne Alard, Aurelie Couturier-Maillard, Olivier Boulard, Denise Boutillier, Myriam Delacre, Carmen Lapadatescu, Annabelle Cesaro, Philippe Blanc, Bruno Pot, Bernhard Ryffel, Mathias Chamaillard, Corinne Grangette

**Affiliations:** 1 Univ. Lille, CNRS, Inserm, CHU Lille, Institut Pasteur de Lille, U1019 - UMR 8204 - CIIL - Centre d’Infection et d’Immunité de Lille, F-59000 Lille, France; 2 INEM - UMR7355, Molecular Immunology, CNRS, University of Orleans, Orleans, France; 3 Present Address: Institute of Infectious Diseases and Molecular Medicine (IDM), and Infectious Diseases, Division of Immunology and South African Medical Research Council (SAMRC) Immunology, Faculty of Health Sciences, University of Cape Town, Anzio Road, Observatory, 7925 Cape Town, RSA; 4 Department of Clinical Immunology, BioProx, Paris, France; 5 Present Address: Institute of Immunology and Microbiology, First Faculty of Medicine, Charles University, Prague, Czech Republic; 6 Present Address: Research Group of Industrial Microbiology and Food Biotechnology, Faculty of Sciences and Bioengineering Sciences, Vrije Universiteit Brussel, Brussels, Belgium; 7 Present Address: Univ. Lille, Inserm, U1003 - PHYCEL - Physiologie Cellulaire, F-59000 Lille, France

**Keywords:** Antimicrobial responses, Applied microbiology

## Abstract

Crohn’s disease is linked to a decreased diversity in gut microbiota composition as a potential consequence of an impaired anti-microbial response and an altered polarization of T helper cells. Here, we evaluated the immunomodulatory properties of two potential probiotic strains, namely a *Bifidobacterium animalis* spp. *lactis* Bl 5764 and a *Lactobacillus reuteri* Lr 5454 strains. Both strains improved colitis triggered by either 2,4,6-trinitrobenzenesulfonic acid (TNBS) or *Citrobacter rodentium* infection in mice. Training of dendritic cells (DC) with Lr 5454 efficiently triggered IL-22 secretion and regulatory T cells induction *in vitro*, while IL-17A production by CD4^+^ T lymphocytes was stronger when cultured with DCs that were primed with Bl 5764. This strain was sufficient for significantly inducing expression of antimicrobial peptides *in vivo* through the Crohn’s disease predisposing gene encoding for the nucleotide-binding oligomerization domain, containing protein 2 (NOD2). In contrast, NOD2 was dispensable for the impact on antimicrobial peptide expression in mice that were monocolonized with Lr 5454. In conclusion, our work highlights a differential mode of action of two potential probiotic strains that protect mice against colitis, providing the rational for a personalized supportive preventive therapy by probiotics for individuals that are genetically predisposed to Crohn’s disease.

## Introduction

Crohn’s disease (CD) is a polygenic form of inflammatory bowel disease (IBD) that affects millions of individuals worldwide. The CD-associated inflammatory lesions are thought to be influenced by environmental factors and have a tendency to develop where the bacterial load is the highest^1^. Since several changes of the gut microbiota composition have been linked to the development of CD^2,3^, antibiotic treatment has been used in some CD patients in order to limit the development of associated harmful bacteria such as pathogenic strains of *Escherichia coli* and many others^4^. However, antibiotic treatment often worsened the underlying bacterial dysbiosis^5^. At present, the therapeutic management of CD remains not curative and far from optimal. Specifically, 25–30% of patients fail to respond to the currently used biologics and/or to immunosuppressive drugs, resulting in accumulation of adverse events, including malignancies and serious infections. An effective prophylactic approach would therefore be extremely important, especially if not accompanied by negative side effects.

CD patients primarily experienced transmural inflammatory lesions at the terminal ileum, where Paneth cells are found more numerous. Clinical studies in CD patients have linked some alterations in the bacterial composition of the gut microbiota and Endoplasmic Reticulum (ER) stress with necroptosis and defects in the antimicrobial activity of Paneth cells^6–8^. Meanwhile, recent genome-wide association studies revealed variants in genes that converge to an impairment of the functionality of Paneth cells^9^. Notably, the protection of stem cells and the secretion of antimicrobial peptides by Paneth cells are impaired in the absence of functional nucleotide-binding oligomerization domain, containing protein 2 (NOD2) - the gene of which is found mutated in more than a third of CD patients of European origin^10–12^. These findings reinforce the notion that decreased secretion of antimicrobial peptides in CD may account for excessive inflammation and necroptosis of Paneth cells through a defective local microbial killing^7^, which is the basis of CD. Given that most current therapies for CD failed to modulate levels of antimicrobial peptides in CD^12^, we investigated whether selected bacterial strains with potential probiotic activities may restore the antimicrobial response protecting mice against colitis.

Multiple studies investigated the use of probiotics to restore the diversity of the gut microbiota composition in order to replace and/or improve conventional IBD therapies^13,14^. Probiotic supplementation was successfully used in preventing pouchitis following ileal pouch anal anastomosis surgery in patients with ulcerative colitis (UC). In contrast, clinical trials with probiotics failed in CD for yet unknown reasons. We have previously shown that selected probiotics exhibit anti-inflammatory capacities in a NOD2-dependent manner^15^. This NOD2-dependent beneficial effect of probiotics could at least in part explain the failure of probiotics administered to CD patients bearing loss-of-function mutations in NOD2. Thus, there is a strong incentive to select probiotic strains that could help to prevent CD development independently of the NOD2 signaling pathway. Particularly, inclusion of additional criteria in the selection procedure should be considered, such as their capacity to induce regulatory T cells (referred herein as Treg) and antimicrobial peptide responses or to strengthen the gut barrier^16,17^.

In the present study, two strains were selected from the Bioprox strain collection, *L. reuteri* 5454 (referred herein as Lr 5454) and *Bifidobacterium animalis* spp. *lactis* 5764 (referred herein as Bl 5764). The aforementioned strains were selected out of fifteen for their capacity to exhibit *in vitro* a strong anti-inflammatory profile (eg. high induction of IL-10 after stimulation of human peripheral blood mononuclear cells, data not shown). We evaluated both *in vitro* and *in viv*o their capacities to alleviate disease severity and their mode of action. Since specific lactobacilli, among which *Lactobacillus reuteri*, have recently been shown to exhibit tryptophan-catabolizing functionality, contributing to aryl hydrocarbon receptor (AHR)-dependent *Il-22* transcription by group 3 innate lymphoid cells^18^, we were also interested to evaluate whether the administration of such probiotic may modulate T helper cells polarization, known to be a critical mediator of mucosal host defense and tissue repair.

In this paper, the anti-inflammatory capacities of strains Lr 5454 and Bl 5764 were demonstrated *in vivo* using a mouse model of 2,4,6-trinitrobenzenesulfonic acid (TNBS)-induced acute colitis and a *Citrobacter rodentium* infection model. Further, the potential of both strains to promote dendritic cell maturation and either IL-17A, IL-22 or FoxP3^+^ regulatory T cells was assessed. Next, the capacity of the selected strains to induce antimicrobial peptides was inspected *in vivo* and we evaluated whether this effect relied on the IL-22/17 signaling pathways and/or on the ability of NOD2 to sense such potential probiotic strains. To this end, germ-free mice, which are deficient in either NOD2, IL-22 or IL-17, were used for a better understanding of the intrinsic potential of the selected strains to induce antimicrobial responses, independently of the gut microbiota.

## Results

### Selected strains improve severity of TNBS-induced acute colitis in mice

**Figure 1:**
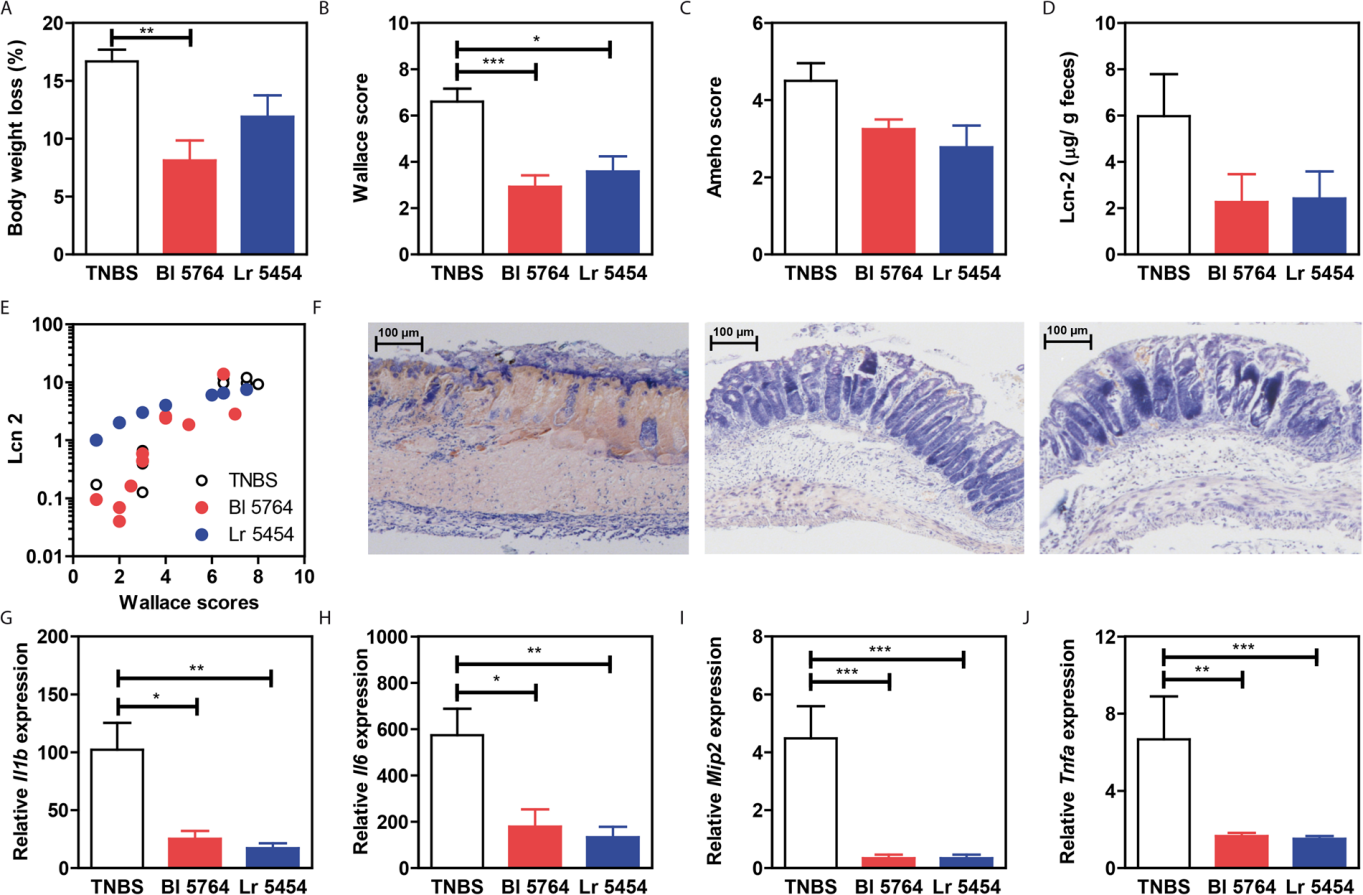
Protective effect of selected strains in a mouse model of TNBS-induced colitis. Individual bacterial strains Lr 5454 and Bl 5764 (5 × 10^8^ CFU of each) or PBS were daily administered for 5 days before and 1 day after TNBS induction to BALB/c mice which were sacrificed 48 hrs after colitis induction. (**A**) Weight loss was evaluated as a percentage of the initial weight at the day of sacrifice; (**B**) Macroscopic description of inflammation (Wallace score). **C**) Histological analyses of inflammation (Ameho score). (**D**) Detection of lipocalin-2 in feces by ELISA and **E**) correlation of lipocalin-2 with Wallace score. (**F**) Representative histology of May-Grünwald stained sections (x10) of the distal colon from mice with acute TNBS-induced colitis that have been administered or not with bacteria. (**G–J**) Colonic gene expression of inflammatory markers followed by qRT-PCR (*Il1b, Il6, Mip2* and *Tnfa*). Typical data of one of the two independent experiments is shown (10 mice per group). The values are expressed as mean ± SEM. *Refers to the comparisons of bacteria-treated groups versus the TNBS control group; *p < 0.05, **p < 0.01, ***p < 0.001.

To evaluate the anti-inflammatory capacity of strains Bl 5764 and Lr 5454, we used a murine model of acute TNBS-induced colitis^19^. Probiotic treatment before colitis induction did not affect the growth of the mice as depicted in Supplementary Fig. 1A. As expected, TNBS-induced loss of body weight and strong macroscopic (Wallace) and histologic (Ameho) scores of inflammation in the colon (Fig. 1A–F). Both strains alleviated colitis symptoms, Bl 5764 being the most efficient, with a significant improvement of body weight loss (p < 0.01) and macroscopic (p < 0.001) score of inflammation (Fig. 1A–C). The results were corroborated strongly with the measurement of fecal lipocalin 2 which was decreased in probiotic-treated mice (Fig. 1D). Importantly, the lipocalin 2 levels correlated with Wallace scores considering all groups of mice (Spearman correlation coefficient r = 0. 9089, p < 0.0001, Fig. 1E) and also for every individual group (control TNBS group: Spearman correlation coefficient r = 0.7212, p = 0.0335; Bl 5764 group: Spearman correlation coefficient r = 0.9291, p = 0.0001; Lr 5454 group: Spearman correlation coefficient r = 1, p < 0.0001). Both strains were able to improve TNBS-induced lesions at the histological level (Fig. 1C,F) and could significantly dampen gene expression of pro-inflammatory mediators in a similar manner (*Il1b*, p < 0.05 and 0.01; *Il6*, p < 0.05 and 0.01; *Mip2*, p < 0.001, *Tnfa*, p < 0.01 and 0.0001, for Bl 5764 and Lr 5454, respectively, Fig. 1G–J). While the IL-17 and IL-22 protein level remained unchanged (Supplementary Fig. 1B,C), induction of colitis by TNBS significantly enhanced the transcription level of *Il22* which was inhibited by both strains (Supplementary Fig. 1D). However, no significant impact was noticed on the transcript level of either *Ahr* or *Il23* (Supplementary Fig. 1E,F).

### **Both potential probiotics significantly alleviate inflammatory responses in the*C. rodentium*infection model**

**Figure 2:**
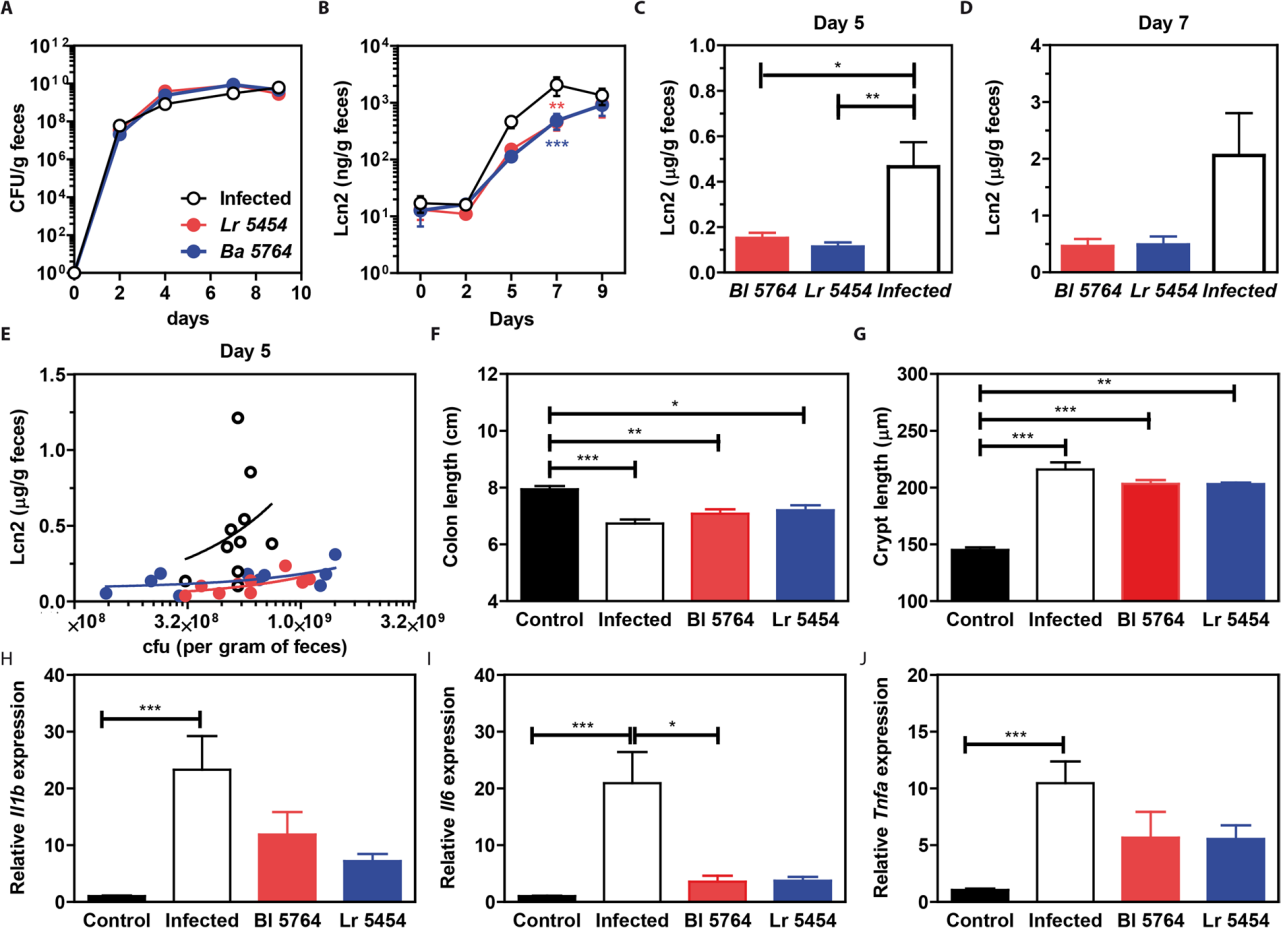
Protective capacities of selected strains in a mouse model of *C. rodentium* infection. Individual bacterial strains Lr 5454, and Bl 5764 (5 × 10^8^ CFU of each) were daily administered 5 days prior to infection until the day of sacrifice to C57BL/6J mice (n = 10 mice per group), while control infected or non-infected (control) mice received only PBS. Mice were orally inoculated with 1 × 10^9^ CFU *C. rodentium* at day 0, sacrificed 9 days after infection and compared to non-infected control mice. (**A**) Bacterial load was assessed in the feces over the course of the experiment. (**B**) Detection of lipocalin-2 in feces by ELISA at different time, (**C**) at day 5 and (**D**) at day 7. (**E**) Correlation of lipocalin-2 with bacterial load at day 5. (**F**) Colon length, (**G**) Crypt length (May-Grünwald and Giemsa stained histology samples) and (**H–J**) gene expression of inflammatory markers (*Il1b*, *Il6* and *Tnfa*) in the proximal colon were evaluated after the sacrifice at day 9. Results are expressed as mean ± SEM. *p < 0.05, **p < 0.01, ***p < 0.001.

To evaluate the anti-inflammatory and possibly anti-infectious capacities of the strains, we next used a mouse model of *C. rodentium* infection, mimicking human infection with enteropathogenic (EPEC) and enterohemorrhagic *Escherichia coli* (EHEC). In comparison to the non-treated control group, the treatment with the two strains had no effect on *C. rodentium* colonization levels, measured at different time points post infection (Fig. 2A). In contrast, measurements of lipocalin2 did show an anti-inflammatory effect of both strains at day 5 and 7 post-infection (Fig. 2B-D). Lipocalin2, as a surrogate marker of inflammation was partially correlating with the load of *C. rodentium* at day five post-infection, even if it failed to reach significance (Fig. 1E). *C. rodentium* infection was characterized by lowered colon length and colonic crypt hyperplasia in comparison to healthy mice (Fig. 2F-G, p < 0.001). Both colon and crypt lengths of mice treated with the bacterial strains were markedly reduced (Fig. 2F-G). The anti-inflammatory effect of both strains was corroborated by a significant downregulation for the expression of *Il1b, Il6* and *Tnfa* at 9 days of infection, as depicted in Fig. 2H-J. No significant differences in either the transcription levels of *Ahr, Il23* and *Il22* or the protein levels of both IL-17 and IL-22 were observed in the colon of any mice (data not shown). Therefore, both commensal strains seemed to markedly improve inflammation in the *C. rodentium* infection model, despite no measurable impact on colonization resistance.

### The selected bacterial strains impact on the polarization of T helper and regulatory cells by modulating the dendritic cell function

**Figure 3:**
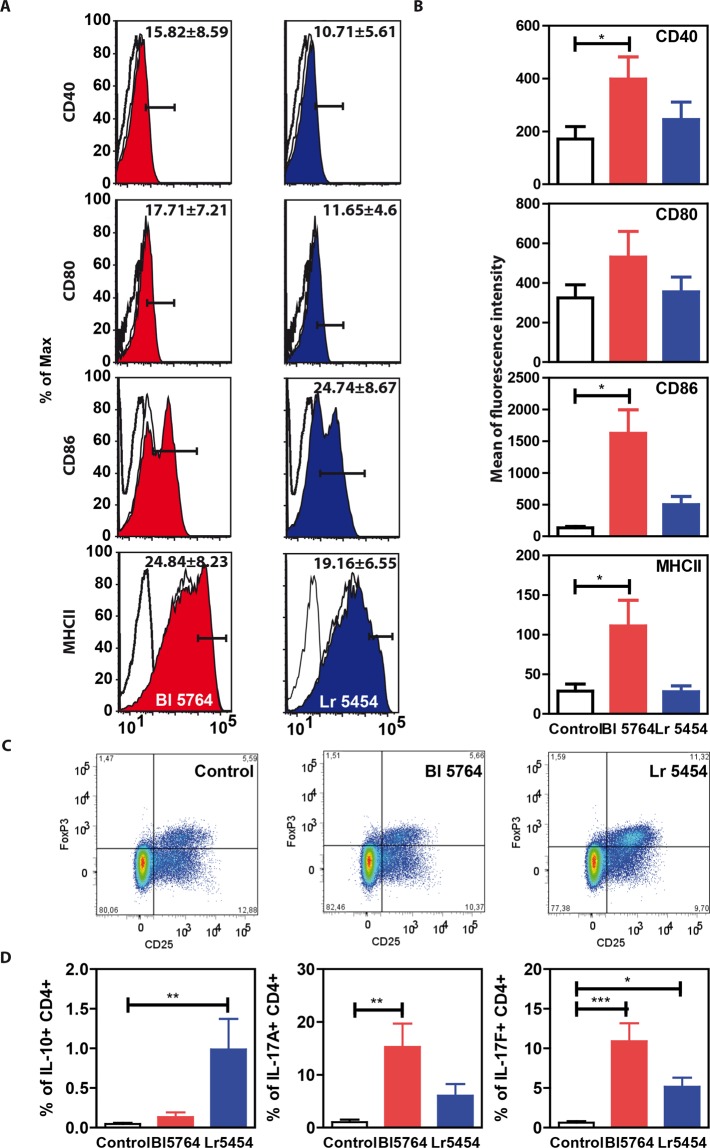
Capacity of the two bacterial strains to activate BMDCs and to promote either Treg or Th17 T cell responses *in vitro*. (**A**) Effect of the two strains on BMDC maturation. BMDCs derived from C57BL/6J mice were stimulated for 24 hrs by bacteria (10:1) and the expression of activation markers CD40, CD80, CD86 and MHCII was followed by flow cytometry. Isotype control is represented by open histogram with dotted line. Expression of activation markers on nonstimulated BMDCs is represented by an open histrogram with solid line. Expression of activation markers on BMDCs stimulated by bacteria is shown by a filled histogram (red for Bl 5764 and blue for Lr 5454). Percentages of positive cells ± SEM are indicated. Representative results of 6 independent experiments are shown. (**B**) Comparison of MFI (Mean of Fluorescence Intensity) of activation markers. Bacteria-primed BMDCs were co-cultured with naïve CD4^+^ T cells (1:10) for 6 days. (**C**) Detection of Tregs (CD4^+^CD25^+^FoxP3^+^) identified from population of CD4^+^ T cells according to the presence of cell surface marker CD25 (PE) and intracellular transcription marker FoxP3 (PE-Cy5) by flow cytometry; representative dot plots with mean and SEM from 6 independent experiments. (**D**) Detection of intracellular cytokines IL-10, IL-17A and IL-17F in CD4^+^ T cells. *Refers to the comparisons of bacteria-treated cells versus untreated cells; *p < 0.05, **p < 0.01, ***p < 0.001.

Besides epithelial sensing, we reasoned that immune responses may also be strengthened through direct activation of dendritic cells and/or group 3 innate lymphoid cells by AHR ligands that could be secreted by these probiotics, as previously reported^18^. To evaluate whether these probiotics may also promote a Th17 response, reflected by IL-17A and IL-22 production, we pulsed bone marrow derived DCs from C57BL/6J mice (Supplementary Fig. 2) with the bacteria for 24 hrs (bacteria/cells ratio of 10:1). The two strains exerted different effects on their maturation (Fig. 3A-B). Notably, high levels of CD40, CD80, CD86 and MHCII were observed in response to strain Bl 5764 in comparison to non-stimulated cells, while no significant induction of all the maturation markers was observed in response to strain Lr 5454 suggesting a different capacity to prime either Treg or Th17 responses (Fig. 3A-B). This led us to further assess the regulatory action of such bacteria by evaluating their impact on the induction of Treg cells that have been found to mediate the anti-allergenic effect of some *L. reuteri* strain^20^. To this end, probiotic-stimulated bone marrow derived DCs were cultured with naïve CD4^+^ CD25^-^ T cells for 6 days (Supplementary Fig. 3) and the phenotype of polarized T cells was then analyzed by flow cytometry (Supplementary Fig. 4, Fig. 3C-D). When compared to Bl 5764, Lr 5454-primed bone marrow derived DCs robustly promoted the differentiation of Treg cells (CD4^+^ CD25^+^ FoxP3^+^), known to regulate *Il-22* expression in CD4^+^ T cells (Fig. 3C). Significant (p < 0.01) increased intracellular presence of IL-10 was also detected in CD4^+^ T cells co-cultured with Lr 5454-primed bone marrow derived dendritic cells while the effect of Lr 5454 was moderate. On the other hand, Bl 5764 significantly favored the increase of IL-17A^+^ (p < 0.01) and IL-17F^+^ (p < 0.001) CD4^+^ T cells (Fig. 3D). The potential capacity of those bacteria to induce Th17 was also followed through the detection of IL-17A and IL-22 in the cell culture supernatants by specific enzyme-linked immunosorbent assay (ELISA). Substantial amounts of IL-17A were found in the supernatants of CD4^+^ T cells derived from C57BL/6 or BALB/c mice, when cultured with respective bone marrow derived dendritic cells that were pulsed with both individual strains even if the effect of Bl 5764 was more prominent (Supplementary Fig. 5B-C). Both strains were also able to induce substantial release of IL-22 (Supplementary Fig. 5A). This was not due to an impact on the proliferation of CD4^+^ T cells, since both strains exerted similar impact on CD4^+^ T cells proliferation (Supplementary Fig. 6). Conversely, no IL-17A and very low IL-22 secretion was detected in the cell culture supernatant of naïve CD4^+^CD25^low^ T cells cultured without BMDCs (Supplementary Fig. 5A-B). This led us to conclude that induction of Treg cells and of Th17 cells is differently regulated through a different intrinsic sensing of these commensals by bone marrow derived dendritic cells.

### **The gut microbiota differentially modulates the impact of*L. reuteri*and*B. animalis*spp**. ***lactis*on intestinal expression of antimicrobial peptides**

**Figure 4:**
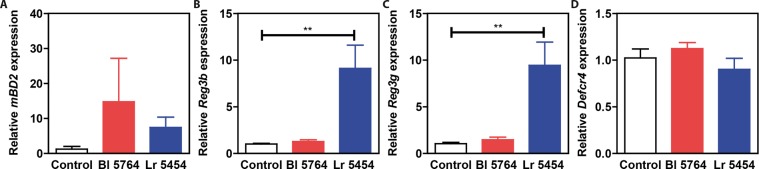
Capacity of Lr 5454 and Bl 5764 bacterial strains to induce antimicrobial responses in naive C57BL/6J mice. Strains (5 × 10^8^ CFU/day/mice) were administered by intragastric gavage to WT conventional C57BL/6J mice for 9 days. Gene expression of *Mbd2, Reg3b and Reg3g* and *Defcr4* was evaluated by qRT-PCR in ileal samples. Values are expressed as the relative mRNA levels compared with samples from untreated mice and represent a mean of 5 mice per group ± SEM. *p < 0.05, **p < 0.01.

**Figure 5:**
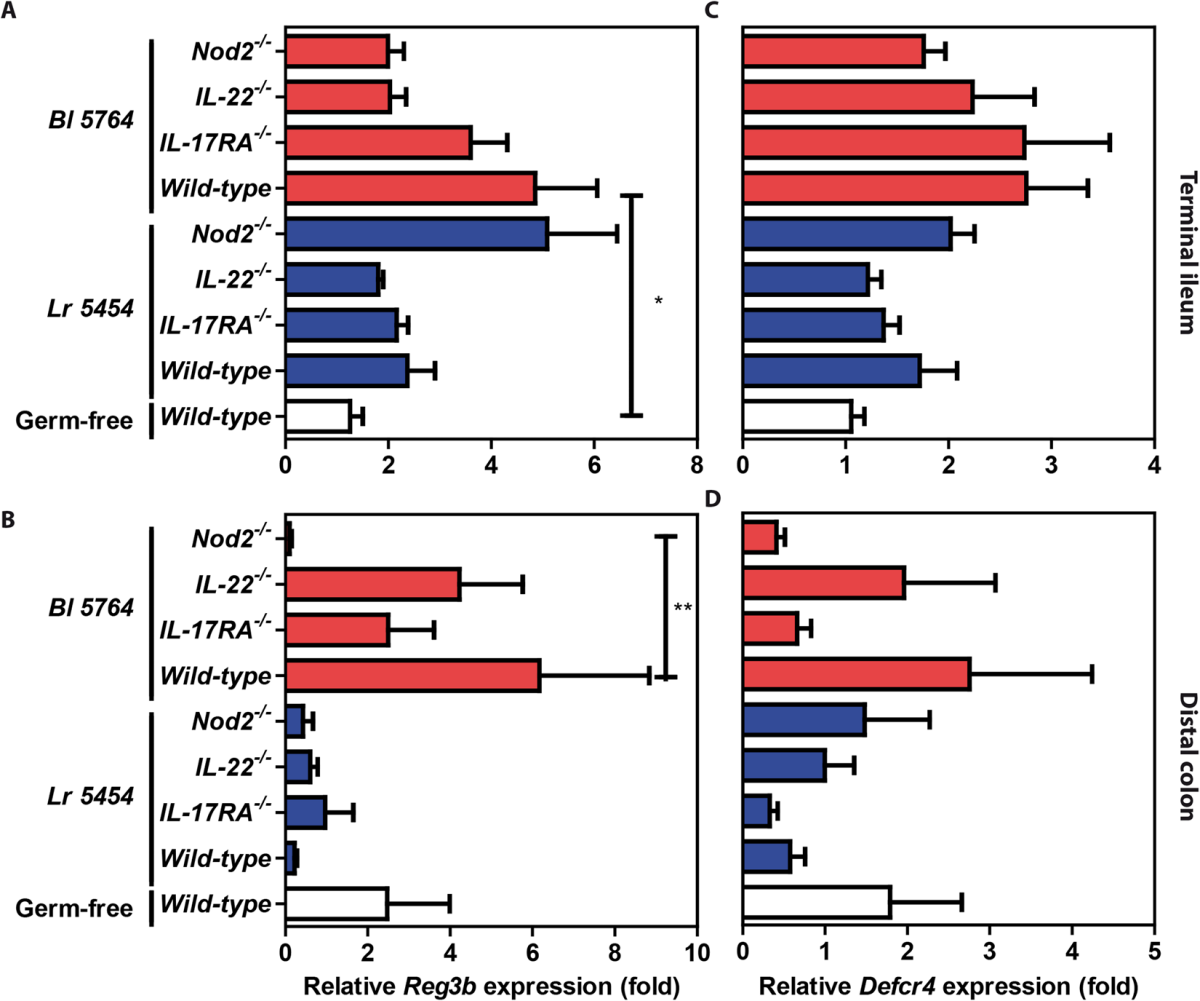
Gene expression of antimicrobial peptides in mono-colonized animals (both WT and KO) supplemented with Lr 5454 and Bl 5764. Bacterial strains (5 × 10^8^ CFU/day/mice) were administered by intragastric gavage to axenic mice derived from C57BL/6J for 9 days. (n = 5 to 9 mice per group). Gene expression of *Reg3b* and *Defcr4* was evaluated by qRT-PCR in (**A–C**) terminal ileal and (**B–D**) distal colon samples, respectively. Values are expressed as the relative mRNA levels compared with ileums from untreated WT germ free mice. Data represent mean values of each group (n = 5–8 mice) ± SEM. *Refers to the comparison of bacteria-treated groups versus untreated germ free mice. *p ≤ 0.05; **p ≤ 0.01.

To ascertain the capacity of the bacterial strains to modulate gene expression of antimicrobial peptides and inflammatory markers, C57BL/6J naive mice were supplemented by Bl 5764 and Lr 5454 for 9 days. On day 10, mice were sacrificed and expression of genes encoding antimicrobial peptides and IL-22 were tested by qRT-PCR analysis. *β-defensin-2 (mbd2)* expression was substantially increased in mice supplemented with Lr 5454 in comparison to those that received Bl 5764 (Fig. 4A). Likewise, the expression of the genes encoding for Regenerating islet-derived protein 3-beta (*Reg3b*) and gamma (*Reg3g*) were significantly (p < 0.01) enhanced in the Lr 5454 group (Fig. 4B-C). Accordingly, the transcript levels of *Il22* were also increased in response to both individual strains, even if this failed to reach significance (data not shown). In contrast, no effect of the bacterial administration on gene expression of *cryptdin 4* was observed (Fig. 4D). To assess whether the effect of the strains relied on accommodating gut microbiota community structure, germ-free mice were mono-colonized with each of the strains. In contrast to what we observed in conventional SPF condition, administration of Bl 5764 significantly enhanced *Reg3b* expression in the terminal ileum (p < 0.05), but to a lesser extent in the colon of monocolonized wild-type mice when compared to germ-free mice. In contrast, colonization with Lr 5454 had no significant effect on its expression in either the small or large intestine of those ex-germ-free mice. The impact of Bl 5764 was significantly repressed in the colon of *Nod2*-deficient monocolonized mice (Fig. 5B). Similar results were observed in the terminal ileum of those animals, even if it did not reach significance (Fig. 5A). The induction of *Reg3b* was also decreased, albeit not significantly, in the gut of *Il-22*- and of *Il17ra*-deficient mice that were mono-colonized with Bl 5764 (Fig. 5A). No significant induction of *cryptdin-4* gene expression were observed in the intestine of mice mono-colonized with both strains (Fig. 5C-D), as observed in conventional mice. Collectively, these observations suggested that the effect of Lr 5454 may result from cooperation with additional commensals for its downstream effect, while the host response to Bl 5764 is particularly impaired by the presence of some microorganisms at the terminal ileum. We could also hypothesize a consequence of the immaturity of the immune system from monocolonized animals. Additional work is still required to evaluate any particular variability of both strains to establish themselves in the gut of either mono-colonized or conventional animals.

## Discussion

In this study, we described the rational and provided criteria for the selection of probiotic strains suitable to prevent the development of colitis and we identified two bacterial strains with promising potential in alleviating intestinal inflammation and modulating antimicrobial peptide expression. Strains Bl 5764 and Lr 5454 indeed improved acute colitis induced by either TNBS or by *C. rodentium*. It is important to emphasize that distinct underlying immune mechanisms can play a role in limiting *C. rodentium* infection, as previously described^21^. During early infection, innate immune mechanisms play a key role and adaptive immune responses occur in a later phase of the infection. Interestingly, distinct probiotic strains and compounds of the microbiota have been shown to affect specific immune mechanisms, capable of downregulating *C. rodentium*-induced inflammation^22^, suggesting that the administration of a mixture of bacterial strains may efficiently collaborate for an adequate maintenance of intestinal homeostasis. In our study, these two probiotics were able to decrease the inflammatory markers Lipocalin-2, IL-1β, IL-6 and TNF-α, as reported already for a *L. acidophilus* strain^23^.

Our work supports the notion that not all commensals exert an immunoregulatory action on Tregs, which have a well-recognized protective role in various inflammatory disorders. We were able to confirm the capacity of Lr 5454 in inducing Tregs in *in vitro* DC/CD4 T cell co-culture, providing a possible explanation for its protective effect against colitis. Such regulatory mode of action pointed out its suitability to be considered in clinical trials in IBD. In contrast, Bl 5764 efficiently induced IL-17A and IL-22, while having a minor impact on Tregs in comparison with Lr 5454. Nevertheless, both strains were able to improve signs of inflammation in both preclinical models, suggesting that such potential probiotic strains may exert their beneficial effects through different mechanisms, as we previously reported^16,17^.

IL-17A has been shown to exert both beneficial^24^ and detrimental effects^25^, but blockage of IL-17 signaling failed to protect against colitis in mice and humans^26^. Indeed the maintenance of gut homeostasis requires IL-17A, that is known to trigger secretion of IL-22 and downstream production of antimicrobial peptides^27^. Both strains clearly induced secretion of IL-22 and IL-17 by CD4^+^ T cells when cocultured with DCs. Antimicrobial peptides are important components of the innate immunity and are often expressed in response to colonic inflammation and infection. *C. rodentium* infection is notably known to induce a subset of antimicrobial responses in the gut which alter the composition of the microbiota and promotes the overgrowth of *C. rodentium* itself. Therefore, we evaluated the capacity of both strains to promote the expression of antimicrobial peptides in naïve mice. We observed a strain-dependent effect, with wide variability. Today, there is only a limited number of studies that evaluated the capacity of candidate probiotic strains to directly induce antimicrobial peptides expression and much of the work has been conducted *in vitro*, notably using the Caco-2 epithelial cell line^28–30^. The effects are time- and dose-dependent and also strain-specific, involving different signaling pathways such as NF-kB, AP-1 and MAPKs. The protective capacity of selected lactobacilli (i.e. *L. gasseri*) in periodontal disease has been correlated with the ability to induce the production of β-defensin in mice infected with *Porphyromonas gingivalis*^31^. In neonatal piglets, the modulation of defense peptides was associated with the up-regulation of the expression of Peroxisome Proliferator-Activated Receptor-γ and G Protein-Coupled Receptor 41^32^. The authors reported spatial heterogeneity patterns of this induction, probably due to the indirect production of butyric acid, mainly triggered in the hindgut after *L. reuteri* administration. The induction of at least some of the antimicrobial peptides could thus be triggered by a complex set of signaling pathways that could explain the observed inter-individual variability. This capacity is of high importance in the context of CD, where impaired defensin expression was observed in most patients^6,33,34^. Careful selection of probiotic strains suitable for CD treatment, is critical since clinical trials using probiotics led to disappointing results in CD so far^13,14^. The potential successful strains should have the capacity to promote the expression of antimicrobial peptides as a potential way to scorrect dysbiosis even in a context of chronic inflammation and the potential presence of several genetic polymorphisms, such as the presence of NOD2 mutations.

The use of mono-colonized animals allowed us to evaluate the role of the gut microbiota community structure on the regulation of antimicrobial response by such potential probiotics. Indeed, the impact of Lr 5454 on antimicrobial peptides relied on the presence of the gut microbiota, while the presence of commensal bacteria exerted a tolerogenic effect on the NOD2-dependent sensing of Bl 5764. Thus, the exact role of the gut microbiota community structure deserves additional experimental studies in order to understand which bacteria and bacteria-derived metabolites may interfere with the induction of antimicrobial peptides in response to these potential probiotics. This is important as the intestine provides a competitive microbial environment^35^ and one can assume that, while additional commensals could be necessary for the optimal growth and function of Lr 5454 through a mechanism of cross-feeding^36^, other species could compete with Bl 5764, as reported for many microbial ecosystems^37^. Alternatively, our studies could help to better understand tolerogenic mechanisms that prevent NOD2-dependent sensing of Bl 5764 in conventional mice. However, it remains to be verified that the results we obtained in mono-colonized mice were not affected by the immature immune system of these mice.

Finally, it is important to remind that between 30% and 50% of CD patients in the Western countries carry *Nod2* mutation^38^. Therefore, identification of strains able to restore gut homeostasis and immune functions in a NOD2-independent manner would be highly desirable. In this study, the use of mono-colonized *Nod2*-deficient mice led us to provide evidence that the selected strain Lr 5454 can trigger NOD2-independent AMP expression. In contrast, an intact NOD2-dependent signaling pathway was required in mono-colonized animals for inducing *Reg3b* expression in response to Bl 5764.

In conclusion, we identified two potential probiotic strains with the capacity to downregulate acute intestinal inflammation that relied on gut microbiota community structure and acting by different downstream mechanisms. Indeed Lr 5454 was unable to induce DC maturation, suggesting as previously described, a better capacity to induce tolerogenic DCs^38^. We indeed demonstrated that this strain was able to preferentially induce Tregs, and *Reg3b* expression in a NOD2-independent manner. In contrast, Bl 5764 had a good capacity to promote bone marrow derived dendritic cells maturation and IL-17A secretion. These potential probiotics could therefore be interesting candidates for further testing in preclinical models of chronic colitis and potentially for human clinical studies to evaluate their combined beneficial effect in the context of different microbiota community structures of CD patients.

## Methods

### **Bacterial strains, culture conditions and reagents**

The two bacterial strains were kindly provided by BioProx. Lactobacilli were grown at 37 °C in MRS broth (Difco, Detroit, USA). Bifidobacteria were cultured in MRS media supplemented with cysteine (0.5 µg/ml) under anaerobic condition. Bacteria were grown overnight, harvested by centrifugation (10 min at 4000 × g), washed twice with PBS buffer (pH 7.2). For *in vitro* stimulation, bacteria were resuspended in PBS containing 20% (v/v) glycerol to a final concentration of 2 × 10^9^ CFU/ml and stored at −80 °C until used. For *in vivo* administration, fresh bacteria or lyophilized powder (provided by BioProx) were resuspended in PBS at 5 × 10^8^ CFU/per mice in 200 µl.

### **Mice**

C57BL/6J mice and BALB/c ByJ (female, 7–8 weeks old) were purchased from Charles River (L’Arbresle, France). Mice were housed in specific pathogen-free condition at the animal facility of the *Institut Pasteur de Lille* (accredited no. C59–350009) and maintained in a temperature-controlled (20 ± 2 °C) environment with a strict 12-hour dark/light cycle. For *C. rodentium* infection, experiments were performed in a biosafety level 2 animal facility. Housing and experimentations were performed in compliance with European guidelines of laboratory animal care (number 86/609/CEE) and French legislation (Government Act 87–848), approved by national and local Animal Ethics Committees (Nord-Pas-de-Calais CEEA N°75, Lille, France) and the *Ministère de l’Education Nationale, de l’Enseignement Supérieur et de la Recherche*, France (accredited no. 201608251651940). Before experimentation, animals were provided a one-week acclimation period and were given *ad libitum* access to regular mouse chow and water.

Germ-free WT and Knock-out (KO) NOD2^−/−^^10^, IL-22^−/−^^39^ and IL-17RA^−/−^^40^ mice on a C57BL/6 background were generated at TAMM/CNRS Orleans using standard protocols and were bred in isolators under strict germ-free conditions.

### Preparation of bone marrow derived dendritic cells (BMDCs) and Treg/Th17 induction

BMDCs were generated from bone marrow progenitor cells of BALB/c and C57BL/6J mice as described previously^15,41^. Briefly, bone marrow progenitor cells were obtained by flushing femur and tibiae of mice. After red blood cell lysis, progenitor cells were seeded at a concentration of 10^5^ cells/ml in supplemented media (RPMI 1640 containing 2mM L-glutamine, 2 mM HEPES, 40 mg/l gentamicin, 10% Fecal bovine serum (FBS), and murine granulocyte monocyte colony stimulating factor (GM-CSF), obtained from the supernatant of a J558 cell line and cultured for 7 days at 37 °C under 5.5% CO_2_. On day 7, the cells were harvested, counted and seeded in a 24-well plate for additional 3 days. The purity of BMDCs evaluated after CD11c labelling is shown in Supplementary Fig. 2 A. On day 10, the cells were stimulated by the bacteria (ratio bacteria/cell: 10:1) in the presence of gentamycin (100 µg/ml). After 24 hrs of stimulation, BMDCs were harvested, washed and stained using mAbs (eBioscience) anti-CD11c PE-Cy7 (clone N418), CD40 PE (clone IC10), CD80 FITC (clone 16–10A1), CD86 PE-Cy5 (clone GL1), MHCII APC (clone MS/114.15.2) and acquired using BD FACS Canto II (Becton Dickinson).

Naive CD4^+^ T cells were obtained from spleens of BALB/c or C57BL/6J mice using the CD4 isolation kit (Miltenyi Biotec). The purity of isolated naïve CD4^+^ T cells was measured after CD4 labelling and the viability was checked using propidium iodide (Supplementary Fig. 2B). Bacteria-primed BMDCs were adjusted to 2 × 10^5^ cells/ml for coculture with naïve CD4^+^ T cells (ratio DC/T cells: 1:10) for 6 days in RPMI media supplemented by FCS 10%, glutamine 2 mM, gentamycin 50 µg/ml. On day 3, Dynabeads Mouse T-Activator CD3/CD28 (Lifetechnologies, 11452D), pan activator of T cells, were added (at a ratio beads/T cells of 2:1) to promote the polarisation impact of probiotic-primed BMDCs on different T cell subsets. The capacity of probiotic-primed BMDCs to affect T cell proliferation was assessed at day 3, using CFSE (carboxyfluorescein succinimidyl ester, 5 μM) proliferation assay by flow cytometry (Supplementary Fig. 2C). After 6 days of co-culture, CD4^+^ T cells were stained using mAbs anti-CD4 FITC (eBioscience, clone RM4–5) and anti-CD25 PE (eBioscience, clone PC61.5). Intracellular staining (permeabilization and fixation) was performed using the Mouse regulatory T cell staining kit, according to the manufacturer’s recommendation (eBioscience). Cells were further stained using the mAbs anti-FoxP3 PE-Cy5 (eBioscience, clone JFK.16 s), anti-IL-17A eFluor 450 (eBioscience, clone 17B7) and anti-IL-17F eFluor 660 (eBioscience, clone 18F10). Samples were acquired using Fortessa (Becton Dickinson).

For IL-17 and IL-22 detection in cell culture supernatants by ELISA, IL-17 Duoset (DY421, R&D System) and IL-22 Duoset (DY582, R&D System), respectively, were used according to the manufacturer’s recommendation.

### Supplementation of naive mice

Strains (5 × 10^8^ cfu/day/mice) were administered by intragastric gavage to WT conventional C57BL/6J or BALB/c mice for 9 days. Samples of ileum were removed at sacrifice and stored in RNAlater® storage solution (Ambion, Life Technologies, Foster City, CA, USA) at −80 °C until qRT-PCR analysis.

### Colitis models

A standardized TNBS-induced murine model of acute colitis was performed using BALB/c mice^42^. Briefly, anesthetized mice received an intra-rectal administration of TNBS (Sigma-Aldrich Chemical, France; 110 mg/kg) dissolved in 0.9% NaCl/ethanol (50/50 v/v). The protective effect of probiotics was evaluated after intragastric administration of bacteria (5 × 10^8^ CFU/mice) starting 5 days before colitis induction. At 48 h after colitis induction, mice were sacrificed by cervical dislocation and colons were removed, washed and opened. Macroscopic inflammation grading was performed blindly using the Wallace scoring method^43^, reflecting both the intensity and the extent of the inflammatory lesions. Histological analysis was performed on May-Grünwald-Giemsa stained 5-µm tissue sections from colon samples fixed in 10% formalin and embedded in paraffin. Immediately after sacrifice, colonic samples were taken and stored in RNAlater® storage solution (Ambion, Life Technologies, Foster City, CA, USA) at −80 °C until qRT-PCR analysis or directly at −80 °C before protein extraction using T-PER tissue protein extraction reagent (Pierce Biotechnology, Rockford, USA). The production of IL-17 and IL-22 was determined as described previously for *in vitro* co-culture experiment.

*Citrobacter rodentium* infection was performed using the kanamycin resistant *C. rodentium* DBS 120 K strain (kindly provided by David Bernard Schauer at MIT, USA). A single colony was cultured overnight in 10 ml Luria-Bertani media (LB) containing 50 µg/ml kanamycin, under agitation. Next day, 20 ml of fresh LB containing 50 µg/ml kanamycin was added and bacteria were cultured for further 6 hrs. Bacteria suspension was centrifuged, washed and resuspended in PBS and adjusted to 5 × 10^9^ CFU/ml. C57BL/6J mice were infected by intragastric administration of *C. rodentium* (10^9^ CFU per mice). To evaluate the potential capacity of the selected probiotic strains, bacteria (5 × 10^8^ CFU per mice) were administered 5 days prior to infection and every following day until day 9 post infection when mice were sacrificed. Levels of infection were monitored at different time points by estimating the bacterial load of *C. rodentium* by plating serial diluted feces homogenates on LB agar containing 50 µg/ml kanamycin and performing CFU enumeration. Immediately after sacrifice, distal colon segments were put in RNAlater® (Ambion, Life Technologies, Foster City, CA, USA) and frozen at −80 °C, until RNA extraction and qRT-PCR analysis.

### Monocolonisation of axenic mice

Germ-free WT and mutant mice (9–13 weeks old) were mono-associated with the potential probiotics by intragastric gavage (5 × 10^8^ CFU/day/mice) for 9 days. Ileal samples were removed at sacrifice and stored in RNAlater® storage solution (Ambion, Life Technologies, Foster City, CA, USA) at −80 °C until qRT-PCR analysis were performed.

### Quantification of fecal lipocalin 2

Freshly collected fecal samples were homogenized using Lysing Matrix D (MPbio, Eschwege, Germany) in PBS containing 0.1% Tween 20 (100 mg/ml). The samples were then centrifuged for 10 min at 12,000 rpm at 4 °C and clear supernatants were collected and stored at −20 °C until analysis, as reported by Chassaing *et al*.^44^. Supernatants were diluted 4-fold until 500-fold depending on severity of the colitis and Lcn-2 levels were measured by ELISA using the Duoset kit (R&D System, Minneapolis, MN, USA), according to the manufacturer’s instructions.

### RNA extraction and quantitative RT-PCR (qRT-PCR)

Tissue samples were homogenized using Lysing Matrix D (MPbio, Eschwege, Germany). RNA from cells and tissue was extracted using Macherey-Nagel NucleoSpin RNAII isolation kit (Düren, Germany) according to the manufacturer’s recommendation. Quantity and quality of RNA was checked by Nanodrop (260/280 nm, 260/230 nm). The 260/280 ratio was higher than 1.95 in all samples. RNA was reverse-transcribed using High Capacity cDNA Reverse Transcription Kit (Applied Biosystems, Woolston Warrington, UK). qRT-PCR was performed using the Power SYBR Green PCR Master Mix (Applied Biosystems) and the ViiA 7 Real-Time PCR System (Applied Biosystems). Primers used are indicated in Table S1. Results are expressed as 2^−∆∆ct^ values as described previously^45^.

### Statistical analysis

GraphPad Prism software was used for graph preparation and statistical evaluation. Statistical significance was determined using non-parametric one-way analysis of variance (ANOVA) followed by Dunn multiple comparison posthoc test and non-parametric two-way ANOVA with Bonferroni post-tests (GraphPad Prism software). Data with p values ≤ 0.05 were considered to be significant. The Spearman correlation coefficient was used to analyze correlation between lipocalin-2 and inflammation scores.

## Supplementary information

Supplementary information.
