# Supplementary material for: Lactobacillus reuteri 5454 and Bifidobacterium animalis ssp. lactis 5764 improve colitis while differentially impacting dendritic cells maturation and antimicrobial responses
Source: Sci Rep. 2020 Mar 24;10:5345. doi: 10.1038/s41598-020-62161-1 (PMC7093418; doi:10.1038/s41598-020-62161-1)
Supplement: Supplementary file 1 — Supplementary information. [file 41598_2020_62161_MOESM1_ESM.pdf]

Lactobacillus reuteri 5454 and *Bifidobacterium animalis* ssp. *lactis* 5764 improve colitis while differentially impacting dendritic cells maturation and antimicrobial responses

Jiří Hrdý[1,4], Jeanne Alard[1], Aurelie Couturier-Maillard[2], Olivier Boulard[1], Denise Boutillier[1], Myriam Delacre[1], Carmen Lapadatescu[3], Annabelle Cesaro[1], Philippe Blanc[3], Bruno Pot[1,5], Bernhard Ryffel[2,*], Mathias Chamaillard[1,6,*] & Corinne Grangette[1,*]

Legend to Supplementary Figures

Supplementary Figure 1. Protective effect of selected strains in a mouse model of TNBS-induced colitis. Individual bacterial strains Lr 5454 and Bl 5764 (5 x 10^8 CFU of each) or PBS were daily administered for 5 days before and 1 day after TNBS induction to BALB/c mice which were sacrificed 48 hrs after colitis induction. A) Weight was followed after probiotic treatment before and after colitis induction. Colon samples were removed 48h after colitis induction and kept frozen at -80°C. After thawing, they were homogenized in T-PER tissue protein extraction reagent and normalized to total protein content for each sample. B) IL-17 and C) IL-22 levels were measured by ELISA in colonic homogenates. D-E-F) Colonic gene expression *of il22, ahr* and *il23* followed by qRT-PCR. Results represent mean levels ± SEM (n = 10 mice per group). $^*p < 0.05$

Supplementary Figure 2. Purity of BMDCs generated from bone marrow precursor cells after 10 day differentiation in the presence of GM-CSF, determined by measuring the level of $CD11c^+$ cells by flow cytometry.

Supplementary Figure 3. Purity and viability of $CD4^+$ T cells. Purity of isolated $CD4^+CD25^-$ T cells used for co-culture experiments was evaluated by measuring the level of $CD4^+$ cells and viability was measured after propidium iodide labelling and flow cytometry.

Supplementary Figure 4. Gating strategy used to follow the induction of Tregs. Firstly, lymphocyte gate was set based on forward (FSC-A) and side (SSC-A) scatter. Then doublets were excluded according to FSC-A and FSC-H characteristics followed by gating CD4+ T cells (FITC conjugated antibody against CD4 marker was used).

Supplementary Figure 5. Bacteria-primed BMDC were co-cultured with naïve $CD4^+$ T cells (1:10) for 6 days. A) Concentration of and IL-22 and B) IL-17 in cell culture supernatants of cells derived from C57BL/6 mice or C) from BALB/c mice. * refers to the comparisons of bacteria-treated cells versus untreated cells; * $p < 0.05$

Supplementary Figure 6. Impact of bacteria-primed BMDCs on the proliferation of $CD4^+$ T cells using CFSE (carboxyfluorescein succinimidyl ester, 5μM) proliferation assay by flow cytometry.

Supplementary Table 1: Forward and reverse primers used in the study for the respective genes encoding β-actin, IL-1β, TNF-α, CXCL-2, IL-6, IL-22, β-defensin 2, Reg-3β, Reg-3γ and Cryptdin 4.

bact-F	5’- CTAAGGCCAACCGTGAAAAC -3’
bact-R	5’- ACCAGAGGCATACAGGGACA -3’
il1b-F	5’- TTGACGGACCCCAAAAGATG -3’
il1b-R	5’- AGAAGGTGCTCATGTCCTCA -3’
tnfa-F	5’- CCCTCACACTCAGATCATCTTCTC -3’
tnfa-R	5’- GGCTACAGGCTTGTCACTCG -3’
cxcl2-f	5’- CAAAAGATACTGAACAAAGGCAA -3’
cxcl2-R	5’- TCAGGTACGATCCAGGCTTCC -3’
il6-F	5’- AGCCAGAGTCCTTCAGAGAGATAC -3’
il6-R	5’- AATTGGATGGTCTTGGTCCTTAGC -3’
il22-F	5’- TTGAGGTGTCCAACTTCCAGCA -3’
il22-R	5’- AGCCGGACGTCTGTGTTGTTA -3’
Bd2-F	5’- AAAGTATTGGATACGAAGCAGAACTT -3’
Bd2-R	5’- GGAGGACAAATGGCTCTGACA -3’
Reg3b-F	5’- ATGCTGCTCTCCTGCCTGATG -3’
Reg3b-R	5’- CTAATGCGTGCGGAGGGTATATTC -3’
Reg3g-F	5’- CTGTGGTACCCTGTCAAGAGC -3’
Reg3g-R	5’- GGCCTTGAATTTGCAGACAT -3’
Crypt4-F	5’- TGGCCTCCAAAGGAGATAGACA -3’
Crypt4-R	5’- AGGCTGATCCTATCCAAAACACA -3’

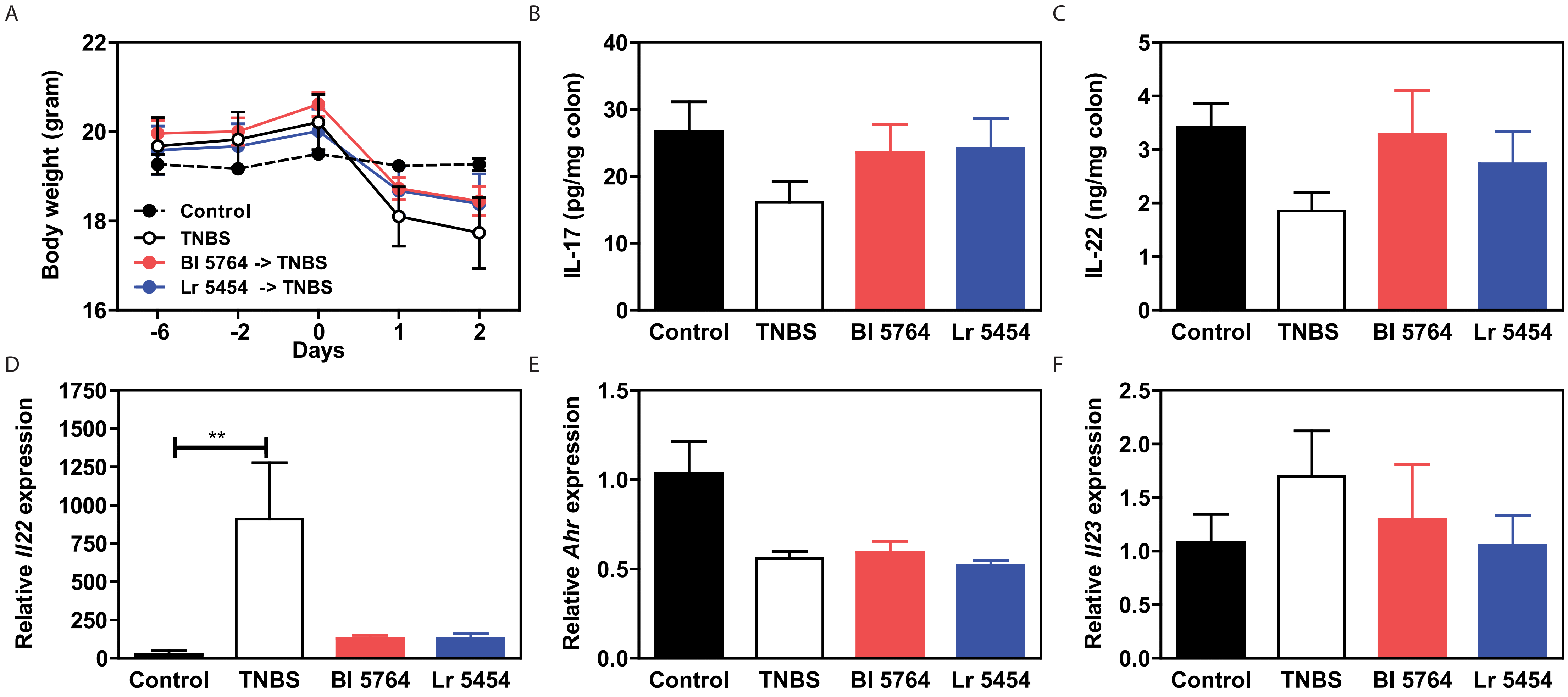
A
B
C
D
E
F
Body weight (gram)
22
20
18
16
-6
-2
0
1
2
Days
Control
TNBS
Bl 5764 -> TNBS
Lr 5454 -> TNBS
IL-17 (pg/mg colon)
40
30
20
10
0
IL-22 (ng/mg colon)
5
4
3
2
1
0
Relative Il22 expression
1750
1500
1250
1000
750
500
250
0
**
Relative Ahr expression
1.5
1.0
0.5
0.0
Relative Il23 expression
2.5
2.0
1.5
1.0
0.5
0.0
Control
TNBS
Bl 5764
Lr 5454

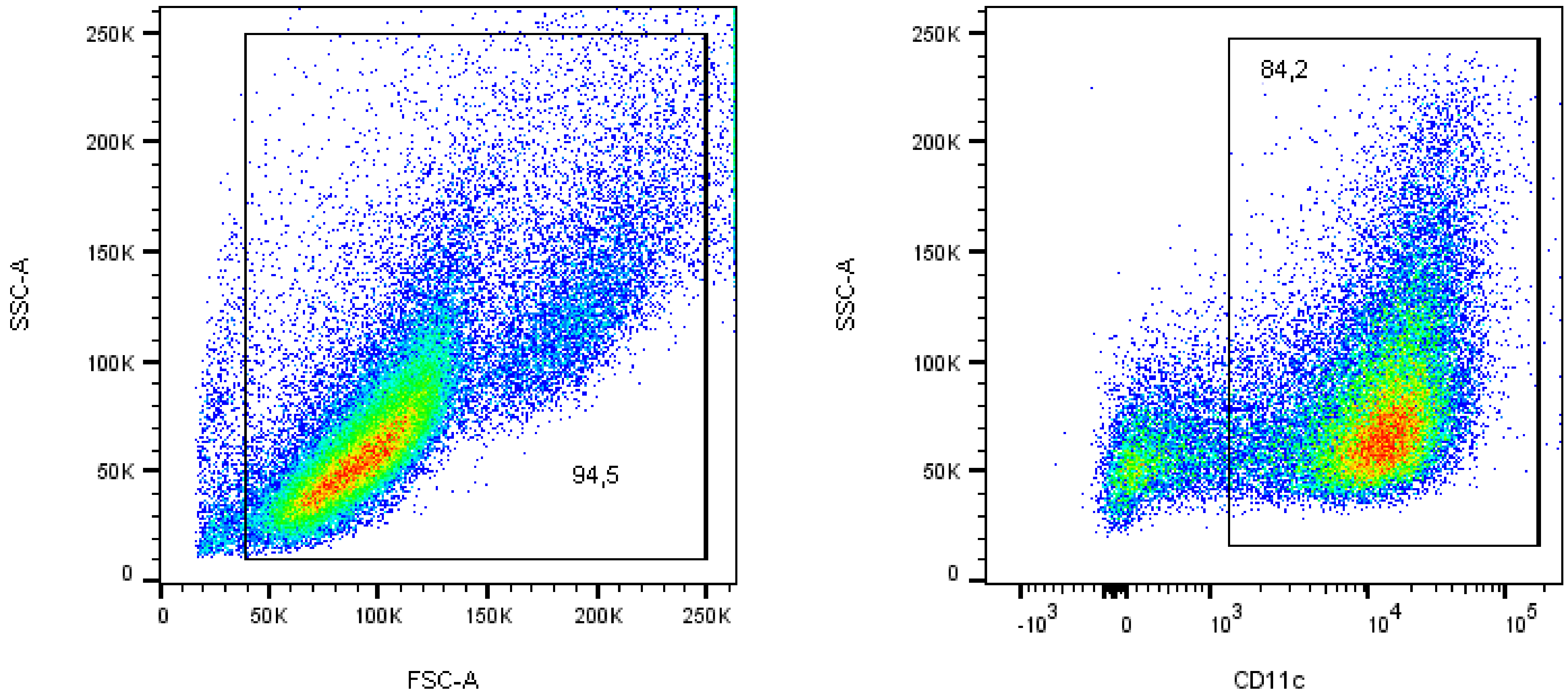
SSC-A
250K
200K
150K
100K
50K
0
0
50K
100K
150K
200K
250K
FSC-A
94,5
SSC-A
250K
200K
150K
100K
50K
0
-10^3
0
10^3
10^4
10^5
CD11c
84,2

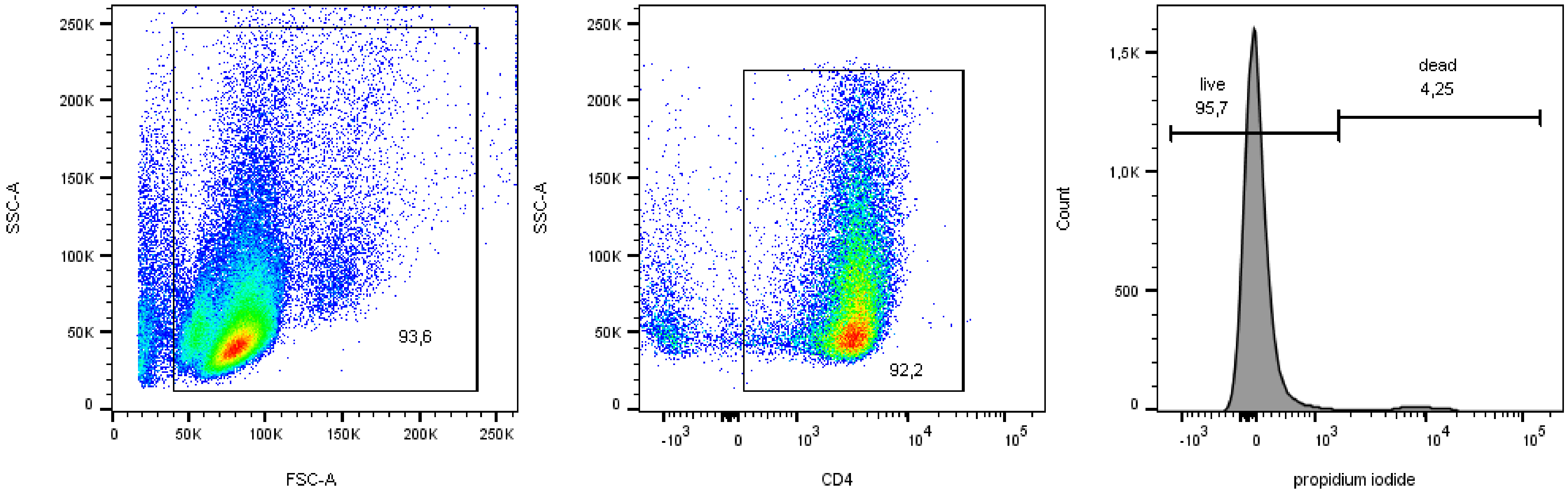

SSC-A
250K
200K
150K
100K
50K
0
0
50K
100K
150K
200K
250K
FSC-A
93,6
SSC-A
92,2
CD4
Count
1,5K
1,0K
500
live
95,7
dead
4,25
propidium iodide

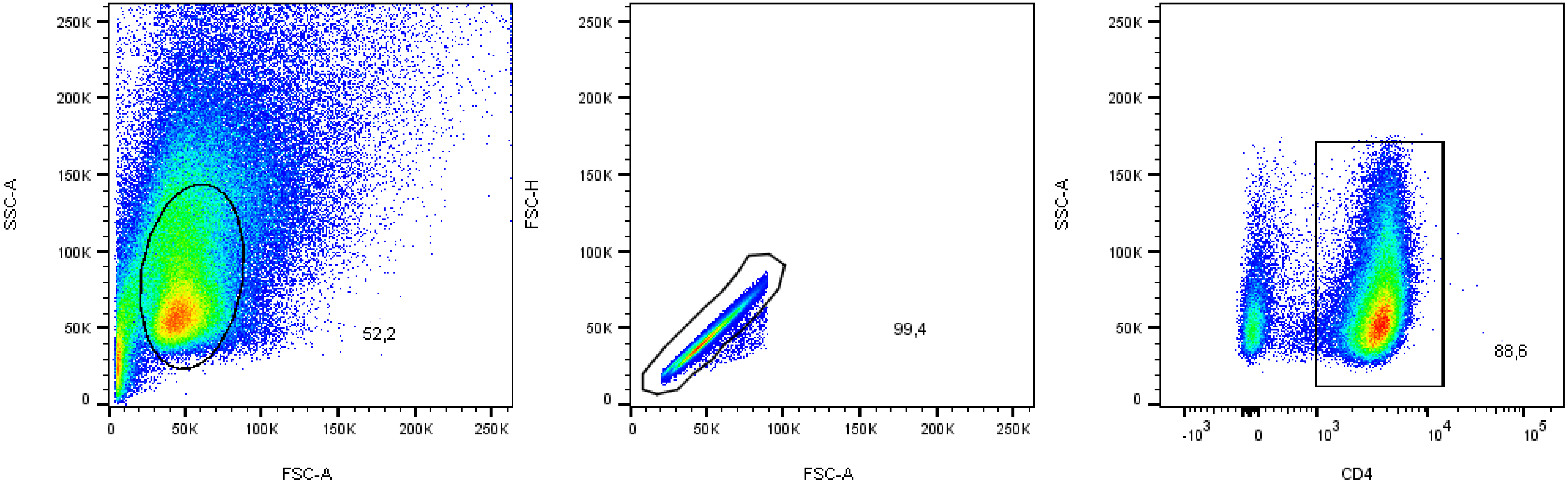
SSC-A
FSC-A
52,2
FSC-H
FSC-A
99,4
SSC-A
CD4
88,6
250K
200K
150K
100K
50K
0
-10^3
10^3
10^4
10^5

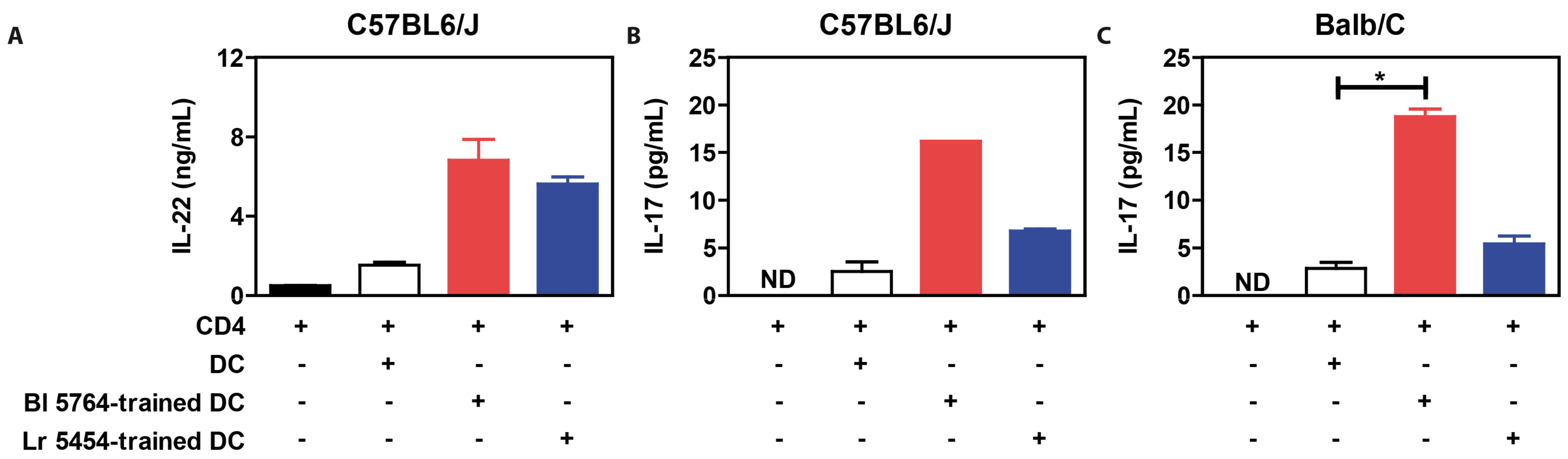
A
C57BL6/J
IL-22 (ng/mL)
0
4
8
12
B
C57BL6/J
IL-17 (pg/mL)
0
5
10
15
20
25
ND
C
Balb/C
IL-17 (pg/mL)
0
5
10
15
20
25
ND
*
CD4
DC
Bl 5764-trained DC
Lr 5454-trained DC
+ + + +
- + - -
- - + -
- - - +

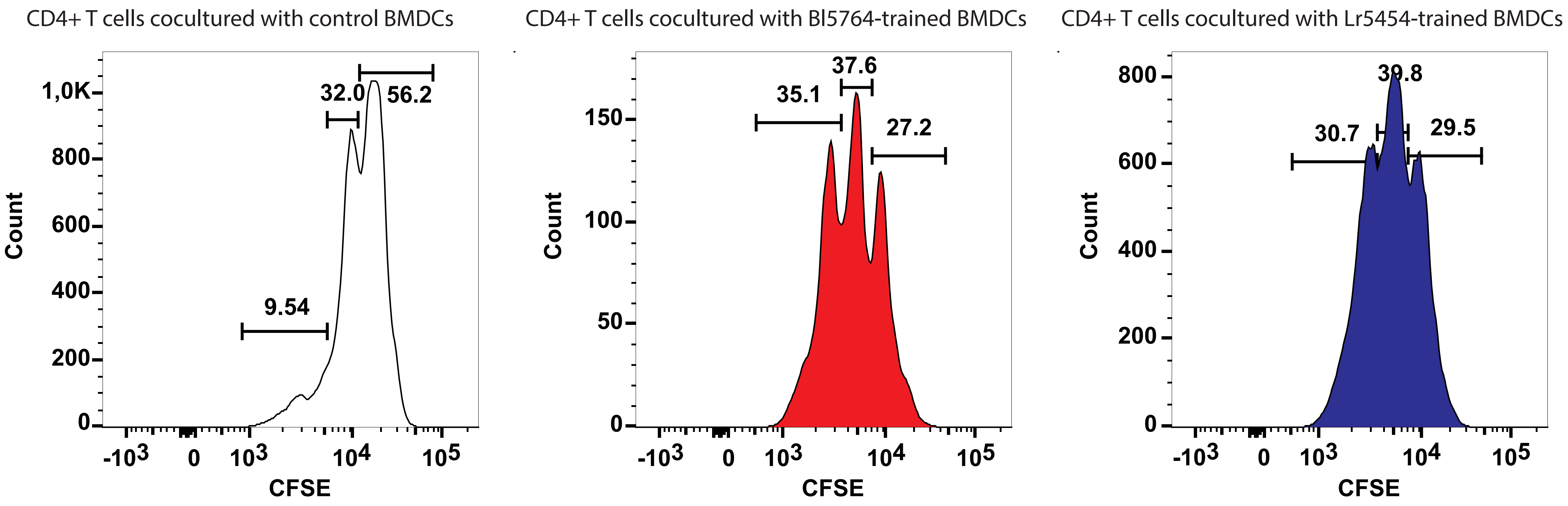
CD4+ T cells cocultured with control BMDCs
CD4+ T cells cocultured with Bl5764-trained BMDCs
CD4+ T cells cocultured with Lr5454-trained BMDCs
Count
CFSE
1,0K
800
600
400
200
0
9.54
32.0
56.2
150
100
50
35.1
37.6
27.2
30.7
39.8
29.5
-10^3
0
10^3
10^4
10^5
